# Irisin promotes osteoblast proliferation and differentiation via activating the MAP kinase signaling pathways

**DOI:** 10.1038/srep18732

**Published:** 2016-01-07

**Authors:** Xiao Yong Qiao, Ying Nie, Ya Xian Ma, Yan Chen, Ran Cheng, Wei Yao Yinrg, Ying Hu, Wen Ming Xu, Liang Zhi Xu

**Affiliations:** 1 Department of Obstetrics and Gynecology, West China Second University Hospital, Sichuan University, Chengdu, People’s Republic of China; 2 The Joint Laboratory for Reproductive Medicine of Sichuan University–The Chinese University of Hong Kong, People’s Republic of China

## Abstract

Physical exercise is able to improve skeletal health. However, the mechanisms are poorly known. Irisin, a novel exercise-induced myokine, secreted by skeletal muscle in response to exercise, have been shown to mediate beneficial effects of exercise in many disorders. In the current study, we demonstrated that irisin promotes osteoblast proliferation, and increases the expression of osteoblastic transcription regulators, such as Runt-related transcription factor-2, osterix/sp7; and osteoblast differentiation markers, including alkaline phosphatase, collagen type 1 alpha-1, osteocalcin, and osteopontin *in vitro*. Irisin also increase ALP activity and calcium deposition in cultured osteoblast. These osteogenic effects were mediated by activating the p38 mitogen-activated protein kinase (p-p38 MAPK) and extracellular signal-regulated kinase (ERK). Inhibition of p38 MAPK by SB023580 or pERK by U0126 abolished the proliferation and up-regulatory effects of irisin on Runx_2_ expression and ALP activity. Together our observation suggest that irisin directly targets osteoblast, promoting osteoblast proliferation and differentiation via activating P38/ERK MAP kinase signaling cascades *in vitro*. Whether irisin can be utilized as the therapeutic agents for osteopenia and osteoporosis is worth to be further pursued.

Osteoporosis is the most common bone metabolic disease characterized by decreased bone mineral density (BMD) and increased risk of fractures^1^,^2^, which is a common comorbidity for sarcopenia^3^. It has become a huge global problem for the aging of population^2^. Physical exercise is recommended as one of the most important non-pharmacological prevention and therapeutic protocols for osteoporosis^4^,^5^. It has been widely assumed that physical exercise exerts anabolic effects on bone either directly through muscle force-generated mechanical signals or indirectly via endocrine regulation^6^. However, the underlying mechanism is unclear. The evidences from basic and clinical research on bone metabolism and muscle biology have revealed the close interaction between muscle and bone in respond to internal and external factors such as aging and mechanical forces^7^.

Although the underlying mechanism and biological processes are unclear, it has been demonstrated that bone structure and mass are closely related to muscular activity, and muscle strength were positively associated with BMDs^8^. Whereas sarcopenia can result in progressive bone loss, bone fragility and higher risk for osteoporosis and fractures^3^. Accumulating evidence suggests that muscle is an endocrine organ, which secretes and releases a range of myokines mediating physical functions and communicating with other tissues and organs^9^,^10^. Recently, endocrine factors secreted by skeletal muscle have been identified as the messengers from muscle to bone during exercise^9^.

The newly identified irisin, an exercise-induced myokine, is cleaved from fibronectin type III domain containing protein 5 (FNDC5) and secreted to serum in response to exercise^11^. Recent research has demonstrated that many metabolic diseases were associated with altered serum irisin level^12^, irisin is considered to mediate the benefits of exercise in these metabolic disorders^13^, including diabetes, obesity, and other related metabolic disorders by promoting the browning of beige fat cells in white adipose tissue, which results in enhanced thermogenesis and increased energy expenditure^14^,^15^. Studies by Andrea Palermo^16^ and Anastasilakis^17^ confirmed that lower irisin level is associated with osteoporotic fractures. Moreover, Colaianni *et al.* found that the conditioned media (CM) collected from myoblasts of exercised mice induced osteoblast differentiation *in vitro*^18^. However, whether and how irisin regulate the bone metabolism is still unclear. Here we demonstrate that irisin can directly targets osteoblast and promotes osteoblast proliferation, differentiation and mineralization *in vitro* via activating the p38 mitogen-activated protein kinase (p38 MAPK) and extracellular signal-regulated kinase (ERK) signaling pathways.

## Result

### Preparation and Identification of irisin from cultured media

**Figure 1:**
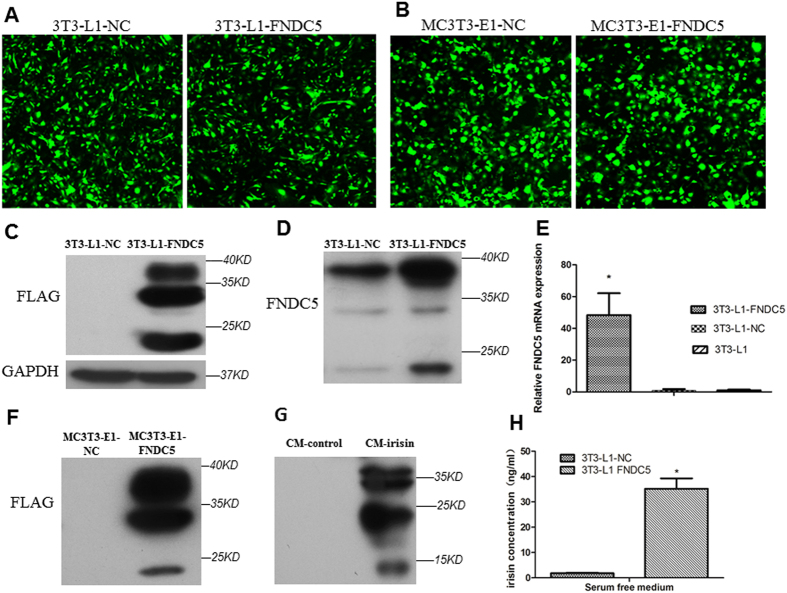
FNDC5 Transfection and Irisin identification. (**A**) Representative images for 3T3-L1-FNDC5 cell (3T3-L1 cell stable express FNDC5-flag and GFP), 3T3-L1-NC cell (3T3-L1 cell stable express GFP as negative control);(**B**) Representative images for MC3T3-E1-FNDC5 cell (MC3T3-E1 cell stable express FNDC5-flag and GFP) and MC3T3-E1-NC cell (MC3T3-E1 cell stable express GFP as negative control). The Flag (**C**) and FNDC5 (**D**) protein expression in 3T3-L1-FNDC5 and 3T3-L1-NC cell lysates were analyzed by Western blotting. The relative mRNA expression of FNDC5 in 3T3-L1-FNDC5, 3T3-L1-NC and 3T3-L1 cell were analyzed by qPCR (**E**). The Flag protein expression in MC3T3-E1-FNDC5 and MC3T3-E1-NC cell lysates were analyzed by Western blotting (**F**). The irisin in CM-irisin (concentrated serum-free medium from 3T3-L1-FNDC5 cell) and CM-control (concentrated serum-free medium from 3T3-L1-NC cell) was verified by Western blot with the antiFNDC5/Irisin antibody (**G**). The concentration of irisin in serum-free medium from 3T3-L1-FNDC5 and 3T3-L1-NC cells was measured by ELISA, The data were expressed as the Means ± SD (n = 3) with 4 replicates (**H**). *P < 0.05 vs.3T3-L1-NC group.

In order to study the role of irisin close to natural state, we used the method described previously^11^. After transduction of the expression lentivirus carring FNDC5-flag/GFP or GFP mRNA only into 3T3-L1cell respectively (Fig. 1A), we found 40-fold increase of FNDC5 mRNA expression level in 3T3-L1-FNDC5 cells (3T3-L1 cell overexpress FNDC5-flag and GFP) compared with 3T3-L1-NC cells (3T3-L1 cell overexpress GFP) as shown in Fig. 1E. Flag-tag protein only can be detected in 3T3-L1-FNDC5 cells, but 3T3-L1-NC cells in western blot (Fig. 1C). Then we obtain CM-irisin by concentrating the serum-free media from 3T3-L1-FNDC5 and CM-control from 3T3-L1-NC using ultrafiltration. On western blot, obvious positive bands were detected in CM-irisin with anti-FNDC5 antibody, the sizes of bands were consistent with previous reports^11^,^19^,^20^. However, no obvious same bands were observed in CM-control (Fig. 1G). In order to analyze the effect in subsequent experiment, we measured the concentration of irisin in serum-free media by ELISA. Our data demonstrated that irisin in 3T3-L1-FNDC5 serum-free media is 35.19 ± 4.10 ng/ml, while irisin in 3T3-L1-NC serum-free media is 1.81 ± 0.20 ng/ml (Fig. 1H). These results confirmed the overexpression of FNDC5 in 3T3-L1-FNDC5 cell line and indicated that our method can obtain irisin in culture medium.

### Irisin promotes osteoblast proliferation

**Figure 2:**
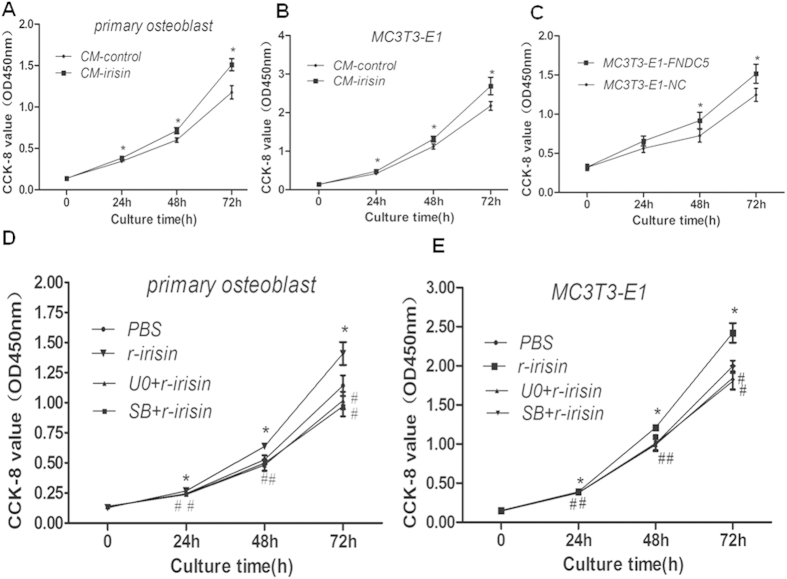
irisin promotes osteoblast proliferation via P38 and ERK signaling pathways. primary osteoblast (**A**) and MC3T3-E1 cell (**B**) were cultured and treated with CM-irisin (irisin 100 ng/ml) or CM-control (irisin < 5 ng/ml). The OD value at 450 nm (OD450 nm) was measured for 72 h, and the data were expressed as the Means ± SD (n = 3) with six replicates. *P < 0.05 vs. the CM-control group at the same time points.(**C**) MC3T3-L1-NC osteoblast and MC3T3-L1-FNDC5 osteoblast were cultured for 3 days after adherence at the same density, the OD450 nm was measured, and the data were expressed as the Means ± SD (n = 3) with six replicates. *P < 0.05 vs. the MC3T3-E1-NC group. Primary osteoblast (**D**) and MC3T3-E1 cell (**E**) were pretreated with PBS as control, U0 (U0126 10 uM for 30 mins) or SB (SB203580 10 uM for 30 mins), then cell was cultured and treated with or with out r-irisin (100 ng/ml), the OD450 nm was measured, and the data were expressed as the Means ± SD (n = 3) with 6 replicates. *P < 0.05 vs. PBS group, ^#^P < 0.05 vs. r-irisin group.

Since osteoblast proliferation is one of the most important indicator of osteogenic effect, we firstly observed the cell proliferation curve of cultured primary rat osteoblast and mouse osteoblastic cell line, MC3T3-E1 cell, by CCK-8 to determine if irisin can promote the proliferation of osteoblast cell. We found that both primary osteoblast and MC3T3-E1 cells treated with CM-irisin began to show higher value of OD450 than the cells treated by CM-control at 24 hours (Fig. 2A,B). The difference could be observed and the OD450 increased more obviously at 48 hours and 72 hours (Fig. 2A,B, Supplementary Table 1). In addition, we observed a similar trend by treat the cells using r-irisin instead of CM-irisin (Fig. 2D,E), and these difference have statistical significance (Supplementary Tables 2 and 3). More Interestingly, we found that MC3T3-E1-FNDC5 cells (MC3T3-E1 cell transducted by FNDC5/GFP expression lentivirus) (Fig. 1B) exhibited increased proliferation compared with MC3T3-E1-NC cells (MC3T3-E1 cell transducted by GFP expression lentivirus) as shown in Fig. 2C, although the difference have statistical significance until 48 h (Supplementary Table 4). These results indicated that FNDC5/irisin directly targets osteoblasts and promotes osteoblast proliferation.

### Irisin promotes osteoblast differentiation and mineralization

**Figure 3:**
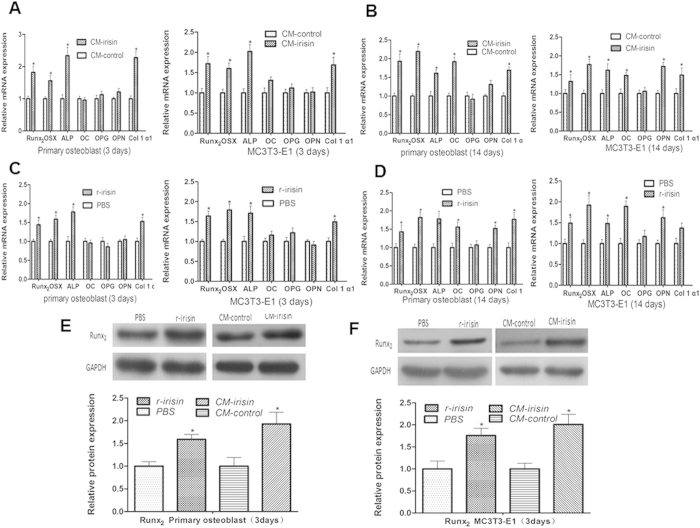
Irisin promotes osteoblast differentiation. primary rat osteoblast and MC3T3-E1 cell were treated with CM-irisin (irisin 100 ng/ml) or CM-control (irisin < 5 ng/ml) in osteogenic differentiation media for 3 days (**A**), 14 days (**B**), or primary rat osteoblast and MC3T3-E1 cell were treated with r-irisin (100 ng/ml) or PBS as control in osteogenic differentiation media for 3 days (**C**), 14 days (**D**), the expression levels of osteoblast transcript regulators (Runx_2_, Osx) and differentiation marker genes (Alp, OC, OPG, OPN and Colα1) were assayed by qPCR. The Runx_2_ protein Level in primary rat osteoblasts (**E**) and MC3T3-E1 cell (**F**) were analyzed by Western blotting. Densitometric analysis of the related bands were expressed as relative optical density of the bands, corrected using GAPDH as control and normalized. The data were expressed as Mean ± SD (n = 3). *P < 0.05 vs. CM-control or PBS group.

**Figure 4:**
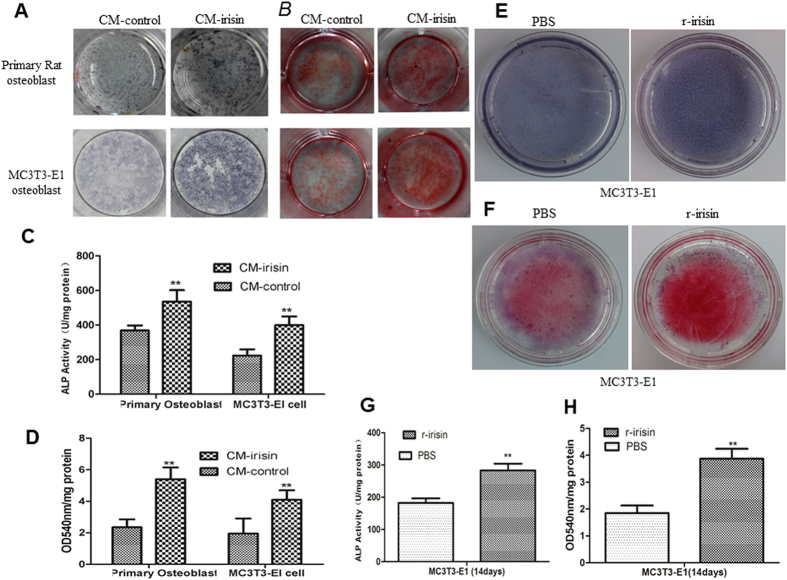
Irisin enhances osteoblast differentiation and mineralization. Representative images of primary rat osteoblast and MC3T3-E1 cell treated with CM-irisin or CM-control by alkaline phosphatase staining (**A**) and Alizarin Red staining (**B**) after culturing in osteogenic medium for 14 days. (**C**) ALP activity in the cell lysate was measured and normalized to the total protein content. The activity was expressed as the Mean ± SD (n = 3). (**D**) Quantification of Alizarin Red S stain via extraction with Hexadecylpyridinium Chloride Monohydrate, The amount of released dye was quantified by spectrophotometry at 540 nm. Representative images of MC3T3-E1 osteoblast treated with r-irisin (100 ng/ml) or PBS as control by alkaline phosphatase staining (**E**) and Alizarin Red staining (**F**) after culturing the cells in osteogenic medium for 14 days, the ALP activity (**G**) and Quantification of Alizarin Red S stain (**H**) was measured and normalized to the total protein content. The data were expressed as Mean ± SD (n = 3).**P < 0.01 vs. CM-control or PBS group.

Many cytokine, such as leptin and adiponectin, can enhance osteoblast proliferation, differentiation, mineralization and bone formation^21^,^22^. Therefore, we analyzed the effects of irisin on osteoblast differentiation and mineralization^23^. Our results demonstrated an increased mRNA expression of osteoblast transcription regulators, including Runt-related transcription factor-2 (Runx_2_) and osterix (Osx, also know sp7), and earlier osteoblast differentiation marker genes, ALP and collagen type 1 alpha-1 (Col1α1). The expression level of these genes increased 1.5–2.3 fold in CM-irisin and 1.4–1.8 fold in r-irisin group than the control after cultured in osteogenic differentiation media for 3 days (Fig. 3A–D). Irisin also increased the expression of later differentiation genes, such as osteocalcin (OC) and osteopontin (OPN) after cultured in osteogenic differentiation media for 14 days (Fig. 3E–H), however, we did not observed any obvious change in osteoprotegerin (OPG) gene expression by treated with either CM-irisin or r-irisin. In addition, we confirmed that the irisin increased the expression of Runx_2_, a key transcription factor associated with osteoblast differentiation^24^,^25^,^26^, on the protein level as shown in Fig. 3E and 3F. Furthermore, a significantly enhanced ALP signal in histochemical staining showed in CM-irisin and r-irisin group (Fig. 4A,E), which also exhibited a markedly greater ALP activity than the control groups (Fig. 4C,G, Supplementary Table 5). Although both CM-irisin and r-irisin can enhance osteoblast differentiation, however, the up-regulatory expression of markers seems more obvious in CM-irisin group than r-irisin group (Table 1). The increased ALP activity in CM-irisin (399.88 ± 49.25 Vs. 223.20 ± 35.23. Fig. 4C) significantly higher than that in r-irisin group (283.06 ± 20.65 Vs. 182.66 ± 13.77 Fig. 4G). The Alizarin Red staining intensity significantly enhanced after treatment with CM-irisin or r-irisin (Fig. 4B,F), and the amount of calcium deposition is also increased in irisin group than control (Fig. 4D,H)). These results indicate that irisin also promotes osteoblast differentiation and mineralization *in vitro*.

### Irisin mediate the osteogenic effects via the P38/ERK MAPK signaling pathways

**Figure 5:**
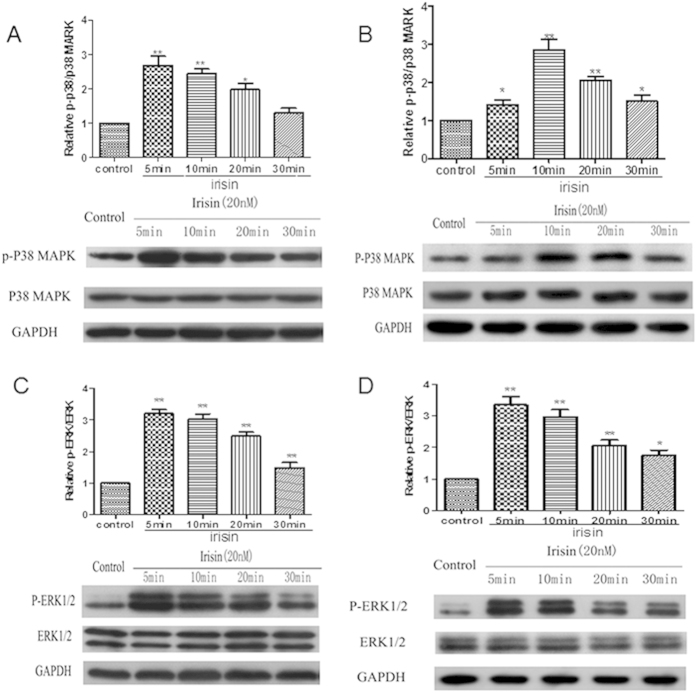
Irisin promotes osteogenetic effect via p38/ERK pathways. Primary rat osteoblast and MC3T3-E1 osteoblast were treated with PBS (control) or r-irisin (20 nM) at indicated time points. Phosphorylated and total p38 and ERK1/2 level in cell lysates were analyzed by Western blotting. Expression levels of P-p38 and total p38 protein in Primary rat osteoblast (**A**) and MC3T3-E1 osteoblast (**B**) were measured by corresponding densitometric quantification. Expression levels of P-ERK1/2 and total ERK in Primary rat osteoblast (**C**) and MC3T3-E1 osteoblast (**D**) were measured with corresponding densitometric quantification. Densitometric analysis of the related bands was expressed as relative optical density of the bands, corrected using GAPDH as control and normalized. The data were expressed as Mean ± SD (n = 3). *P < 0.05; **P < 0.01 vs. control group.

To our knowledge, the irisin receptor has not yet been identified until now. To further elucidate the mechanism of irisin in osteogenesis, we performed signaling pathway experiments to analyze the underlying mechanism. Our data showed that the P38/ERK MAP kinase signaling pathways may play a key role in irisin-induced osteogenesis. A significantly increased amount of phosphorylated P38 (p-P38) and phosphorylated ERK (p-ERK) in both primary rat osteoblast and MC3T3-E1 cell was detected by western blot from 5 minutes to 30 minutes after treatment with irisin, with the peak occurring between 5 and 20 minutes, this effect began to decrease after treatment with r-irisin for almost 30 minutes, while the amount of total P38 and ERK did not change. The increased phosphorylation of P38 and ERK was statistically significant as shown in Fig. 5.

**Figure 6:**
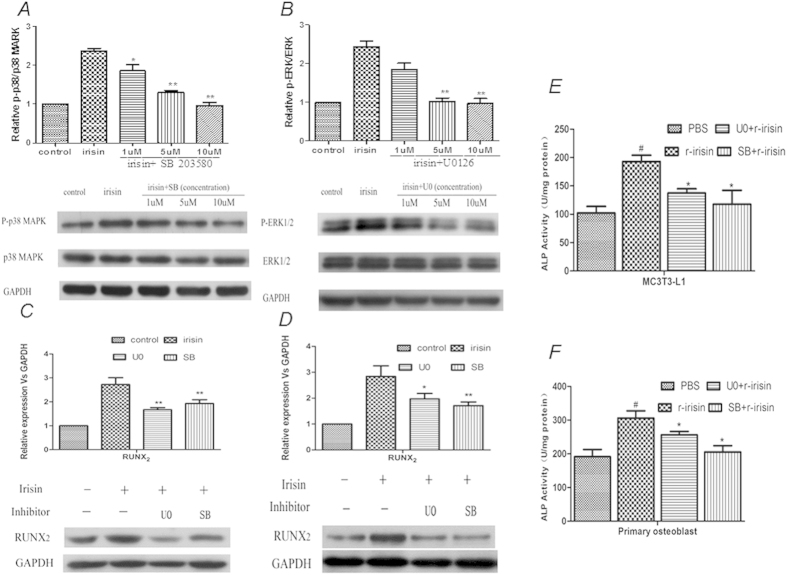
Inhibiting the p38/ERK pathways prevent the irisin-induced upregulatory expression of RUNX_2_ and ALP activity. Primary rat osteoblasts were pretreated with PBS (control), P38 inhibitor SB203580 (SB), or ERK inhibitor U0126 (U0) at the indicated concentrations for 30 minutes, followed by r-irisin (20nM) treatment for 20 mins, phosphorylated and total P38 (**A**) and ERK (**B**) proteins was detected by western blotting. Primary rat osteoblasts (**C**) and MC3T3-E1 osteoblasts (**D**) were pretreated with SB (10 uM) or U0126 (10 uM) for 30 minutes, then, r-irisin (20** **nM) was added to the media for an additional three days. Runx_2_ protein levels were analyzed by Western blotting, The results of the densitometric analysis was corrected and normalized using GAPDH as a control. The data were expressed as the Mean ± SD (n = 3). *P < 0.05; **P < 0.01 vs. control.(**F**) the ALP activity was also measured and normalized to the total protein content. The activity was expressed as the mean ± SD (n = 3), ^#^P < 0.05 vs. PBS group, *P < 0.05 vs. r-irisin group.

To further verify the role of the P38 and ERK signaling pathways in irisin-induced osteogenic effects, osteoblasts were pretreated with PBS (control), or the p38 inhibitor SB203580 (SB), or the ERK inhibitor U0126 (U0) for 30 mins, then cell was cultured in the presence of r-irisin, and P38 and ERK phosphorylation were determined. The result shows that with increasing concentrations of SB and U0, the levels of p-P38 and p-ERK were gradually suppressed, whereas the amount of total P38 and ERK did not change (Fig. 6A,B). This inhibitory effect was statistically significant, as quantified by densitometry. Meanwhile, when osteoblasts were pretreated with SB or U0, the irisin-induced Runx_2_ upregulation (Fig. 6C,D) and increased ALP activity was significantly reduced too (Fig. 6E,F, Table 1). We further analyzed the effects of P38 and ERK signaling pathways in osteoblast proliferation and observed either SB or U0 inhibited the irisin-induced osteoblast proliferation effect (Fig. 2D,E). Finally, we analyzed other well-known signaling pathways reported in many bone studies, including canonical WNT, AKT, and JNK. The phosphorylation level of these proteins were unchanged in our data. (Supplementary Fig. 1). These results indicated that these signaling pathways may not the main signaling pathways involved in irisin-induced osteogenic effect. In conclusion, our results demonstrated that irisin promotes osteoblast proliferation and differentiation via the P38 and ERK signaling pathways.

## Discussion

Osteoporosis is the most common bone disease. Exercise provides clear beneficial effects for the prevention and treatment of osteoporosis, although the mechanisms are poorly understood^27^. Here, we report that irisin, a novel identified exercise-induced myokine, could directly targets osteoblasts and promotes proliferation, differentiation, and mineralization though P38/ERK MAPK signaling pathways.

Our study confirmed that both CM-irisin and r-irisin can directly target osteoblast and enhance osteoblast proliferation. This direct effect indicated that bone is the target organ for irisin, and this effect is not mediated by other tissue, including brown adipose tissue and other factors. In fact, many osteogenic factors, such as estrogen, leptin, adiponectin and so on, can strengthen the bone formation by promoting osteoblast proliferation^21^. Irisin is secreted by skeletal muscle in response to exercise, irisin may promote bone formation so that bone can better adapt to the increased load during persistent exercise. It is clear that both type of irisin have proliferation effects. our result seems CM-irisin have stronger physiological effect than r-irisin, although we can’t draw definitive conclusion only based on present study for the different control groups.

Although the original effect of irisin was to promote adipocyte transdifferentiation and energy metabolism^11^,^14^, irisin-induced proliferation effect was also observed in other cells, including mouse H19-7 hippocampal cells^28^, bone marrow stromal cells^18^, and human umbilical vein endothelial cells^29^. Our data expand our knowledge of irisin in proliferation of cell. Interestingly, the MC3T3-E1-FNDC5 cells also exhibited increased proliferation activity compare with MC3T3-E1-NC cell, this result further confirmed that bone is the target organ of irisin. And furthermore, recent study have indicated that bone also express irisin^30^, irisin may also as an autocrine cytokine mediate physical function in autocrine manner in bone, as irisin, which mainly express in skeletal muscle, can promote myocyte itself metabolism^31^.

We tested the differentiation effects of irisin by treating osteoblast with CM-irisin or r-irisin in osteogenic differentiation media for 14 days. Our results showed that irisin can increased the expression of Runx_2_, which effectuates the expression of bone-specific genes, such as Osx, Col1a1, osteocalcin and so on, by binding to the promoters of these genes. Although the mechanism is still unknown and expected to further study. Runx_2_ is probably one of the targets mediating osteogenic effects of irisin. Our results further confirmed previous findings by Colaianni *et al.*^18^, who showed that conditioned media collected from myoblasts of exercised mice increase the differentiation of bone marrow stromal cells into mature osteoblasts *in vitro*, and our findings are also in agreement with recent evidence showing the association between reduced irisin levels and osteoporotic fractures *in vivo*^16^,^17^. irisin promotes the browning of beige fat cells in white adipose tissue^11^, reduces preadipocyte differentiation, and regulates a variety of factors^13^,^14^, however, brown adipose tissue have osteogenic effects, the experiment *in vivo* is difficult to rule out the indirect effects mediated by the increased brown fat and the resulting molecule. Our study confirm that irisin directly target osteoblast and promote osteogenic effects rather than indirectly effects by browning white adipose tissue or other ways. The irisin-induced osteogenic effects suggest that irisin may be used as a possible treatment target for osteopenia and osteoporosis in future.

Although more than 3 years after the discovery of irisin, the irisin receptor has not yet been identified until now. Elucidating the signaling pathways will be of great significance for the mechanism and application. Our findings demonstrated irisin promote osteoblast proliferation and differentiation via activating the phosphorylation of P38/ERK MAP kinase signaling pathways, and inhibiting phosphorylation of P38/ERK can abolish these effects, we suggest that the P38 and ERK signaling pathway play a critical role in the osteogenesis of irisin. P38 and ERK signaling pathway is very important in cell proliferation and differentiation^21^. MAP kinase signaling maybe the main pathway mediating the effects of irisin, irisin can stimulates browning of white adipocytes through p38 and ERK MAP Kinase Signaling^20^, and promotes human umbilical vein endothelial cell proliferation through the ERK signaling pathway^29^. Our result is in accordance with Colaianni’s latest study, which found that irisin promoted bone osteogenic differentiation of marrow stromal cells via ERK signaling pathway^30^. Erk is one of the important pathway of cell proliferation and differentiation, many molecules inhibit osteoblastic differentiation after activating ERK signaling pathway, while other promote osteoblastic differentiation^32^,^33^,^34^,^35^. We found irisin activated ERK signaling pathway in osteoblast, although the mechanism is unclear, it may be associated with irisin receptor and the way of activation.

Many cytokine and hormones, such as leptin, adiponectin, estrogen and so on, can regulate osteoblast proliferation and differentiation though different signaling pathways, including some well-known pathways, e.g. WNT, AKT and JNK pathway. However, for these signing pathways, our data failed to find obvious changes of phosphorylated and total protein level. These signaling pathways may not be involved in the osteogenic effects mediated by irisin. It is worth noting that we only observed the effects of phosphorylation within one hour after treatment with irisin, we can’t rule out irisin could activate these pathways in a longer time.

Irisin is the secretory portion of FNDC5 protein, which has two N-glycosylation sites. For this reason, irisin is also regarded as a glycoprotein with two glycosylated site^11^,^19^,^36^,^37^. Recent study have reported that the posttranslational glycosylation of the secreted irisin enhances the biological function^20^, however, most of commercial r-irisin from Escherichia coli, including the irisin used in our study, is non-glycosylated irisin. It may not reflect the function in natural state exactly^37^. Therefore, in our study, we observed the function of both r-irisin and CM-irisin at a concentration of 100 ng/ml, which is close to the physical concentration in human serum reported in most studies^38^,^39^,^40^,^41^. In fact, the glycosylation of FNDC5 is different in different cells (Supplementary Fig 2), whether these differences may affect the generation and it’s function of irisin need further research. However, it seems the CM-irisin was more effective than r-irisin in our study, although we can’t draw the exact conclusion for the different control groups in experiment. The glycosylation of irisin may enhance its biological activity. It is noteworthy that due to the limitation of our experiment, we can’t exclude the possibility that the difference was due to mechanism other than altered glycosylation or synergistic effect with other substance in culture media.

Physical exercise has widely recognized benefits on skeletal health^7^, and used as an important protocol for prevention and treatment of osteoporosis^4^,^8^. Exercise can strengthens bone, while disuse and limited movement for severe illness, cachexia and muscular diseases always cause bone loss and increased fracture risk^3^,^6^. However, not all types of exercise benefits bone health, moreover, not all types of exercise can increase the concentration of irisin^42^,^43^. Many studies have found that resistant, anaerobic and high workload straining can increase irisin concentration and benefits bone health^38^,^39^,^44^, while aerobic exercise and low workloads training do not change the irisin concentration^40^,^42^,^43^ and have limited or even no effect on bone health^40^. In combination with our results, irisin is probably the important transmitter, by which exercise and muscle modulates bone metabolism.

Taken together, our data extend our understanding of the role of irisin in osteogenesis metabolism, and provide direct evidence that irisin can be used as an important bone regulate factor, which directly target osteoblast and regulate bone metabolism. Moreover, our study add new clue to the mechanism how exercise strengthen the bone formation. irisin could be great potential application in osteoporosis.

## Materials and Methods

### Reagents

Antibodies against FNDC5 (amino acids 50–150), Runx_2_, were purchased from Abcam (Cambridge, MA, USA). Anti-FLAG, Anti-GAPDH, and HRP-conjugated secondary antibodies were obtained from Zen-Bioscience Company (Chengdu, China). Antibodies against phospho-Akt (Ser473), phospho-Akt (Thr308), Akt, ERK, p-ERK, P38, p-P38, JNK, p-JNK, GSK-3β, p-GSK-3β, and β-catenin were purchased from Cell Signaling Technology (Waltham, MA, USA). dexamethasone, ascorbic acid, β-glycerophosphate and Hexadecylpyridinium Chloride Monohydrate were purchased from Sigma (Louis, MO, USA). r-irisin (067-16) and irisin-ELISA Kit (EK-067-29) were purchased from Phoenix (Burlingame, CA. USA).

### Osteoblast culture

#### Primary rat osteoblast culture

Primary osteoblasts were isolated using a method described previously^45^ and modified slightly. Briefly, calvaria were isolated from eight-week-old female Sprague Dawley rats. After the calvaria were shredded, the bone pieces were sequentially digested five times with 0.1% collagenase II mixed with 0.25% trypsin for 20 minutes. The first two digests were discarded, and the cells obtained from the third to fifth digestions were collected, resuspended, and plated in αMEM media (Gibco, Grand Island, NY, USA) supplemented with 10% fetal bovine serum (Gibco) and 1% penicillin-streptomycin (Gibco). Cells were plated in 6-cm dishes and cultured in a humidified atmosphere of 95% air and 5% CO_2_. The cell culture media was changed every 2–3 days. The primary rat osteoblasts were identified by ALP and Alizarin Red staining after osteoblastic differentiation culture. The cells from the third to sixth passages were used for all experiments.

This study protocol were approved by the Ethics Committee of West China Second University Hospital, Sichuan University, China and complied with the china guidelines for the use of laboratory animals, which conform to the State Scientific and Technological Commission of china published in 1988.

#### MC3T3-E1, MSC and3T3-L1cell line culture

Murine osteoblastic MC3T3-E1 cells (Bioleaf, Shanghai, China) was cultured in α-MEM complete media. 3T3-L1 cells (Bioleaf, Shanghai, China) and mouse bone marrow mesenchymal stem cells MSC (Bioleaf, Shanghai, China) were cultured in DMEM (Gibco, Grand Island, NY, USA) supplemented with 10% fetal bovine serum and 1% penicillin-streptomycin at 37.0 °C in a humidified atmosphere of 95% air and 5% CO_2_. The cell culture medium was changed every 2–3 days.

### Virus transfection and Preparation of irisin from cultured media

The FNDC5 (NM_027402) expression lentivirus with a c-terminal Flag-tag and negative control lentivirus were purchased from Genechem (Shanghai, China). 3T3-L1 cells were plated in 6-well plates, and cells were either transfected by standard methods or transduced with an overexpression lentivirus or a negative control. Briefly, the medium was changed to a culture medium without antibiotic. The lenti-FNDC5 (lentivirus overexpressing FNDC5-3FLAG) or lenti-NC (lentivirus negative control) stocks were added in transfection medium at a multiplication of infection (MOI) of ten, as indicated by the manufacturer’s instructions. Then, 5 μg/mL hexadimethrine bromide (Polybrene, Sigma-Aldrich) was added to improve lentiviral vector transduction efficiency. After 72 hours, the green fluorescent protein (GFP) expression was examined by fluorescence microscopy and the media was changed to a complete growth media with 4 μg/mL puromycin (Sigma-Aldrich). Transduced cell clones were selected until the control cells (3T3-L1 cells) were completely dead. Finally, 3T3-L1-FNDC5 (stable FNDC5 overexpression) cells and 3T3-L1-NC (negative control) cells were obtained. FNDC5 expression was verified by q-PCR and Western blot analysis, while FLAG expression was identified by Western blot analysis.

The 3T3-L1-FNDC5 and 3T3-L1-NC cells were plated at a density of 3 × 10^5^ cells/mL with complete culture medium. Then, the culture medium was removed after culture overnight and the cells were washed three times with phosphate-buffered saline (PBS) and incubated in serum-free DMEM media. The media was collected and changed every 48 hours, the collected media was centrifuged three times at 3,000 rpm and filtered with a 0.22μ m filter, then irisin in the media was concentrated by ultracentrifugation with Amicon Ultra-15 3 kD (Millipore, Ireland) at 4,000 g for 40 minutes at 4 °C. The concentrated irisin stock (CM-irisin) from 3T3-L1-FNDC5 cell serum-free media and the concentrated control stock (CM-control) from 3T3-L1-NC cell serum-free media were stored at −80 °C after verification by Western blot. While the stock was used for all experiments unless otherwise indicated, the stock was added to the culture media at the final concentration of 100 ng/ml, which is close to the physiological concentrations in human serum reported by many studies and was reported by many experiments *in vivo* and *in vitro*.

### Cell proliferation assay

The proliferation activity was measured by the Cell Counting Kit-8 (CCK-8) (Dojindo, Japan). Osteoblast cells were seeded into a 96-well plate at 2 × 10^4^ cells/cm^2^. Then, the medium was removed after adherence and the r-irisin or CM-irisin and control (PBS or CM-control) was added. The cells were continuously cultured for 72 h. After 10 μL of CCK-8 solution was added to each well containing 100 μL of medium and incubated with cells for 2 h, the absorbance was measured at 450 nm. All experiments were performed in six replicates.

### Osteoblast differentiation

Primary rat osteoblasts and MC3T3-E1 cells were cultured in osteogenic medium as described previously^46^. Briefly, cells were cultured at 5 × 10^4^ cells/cm^2^ in osteogenic medium with or without 100 ng/ml irisin, which contains10% FBS, 0.1 μM dexamethasone (Sigma, St. Louis, MO, USA), 10 mM β-glycerophosphate (Sigma, St. Louis, MO, USA), and 50  μg/mL ascorbic acid (Sigma, St. Louis, MO, USA) for fourteen days. At 3, 14 days, cell were harvested for qPCR analysis or western blotting. Cell was fixed and ALP and Alizarin red staining at 14 days.

### Alkaline phosphatase (ALP) staining and assay

Cells were washed twice with PBS, fixed with 4% paraformaldehyde for 10 minutes, rinsed with deionized water, and stained with a BCIP/NBT alkaline phosphatase color development kit (Beyotime, China) for one hour under protection from direct light according to the manufacturer’s instructions, then, images were obtained with a Canon camera. After osteoblasts were cultured in osteogenic medium for fourteen days, the cells were washed and lysed. The cell lysates were centrifuged and the supernatants were used for the alkaline phosphatase assay according to the manufacturer’s protocols (Sigma-Aldrich, Louis, MO, USA). The results were calculated and normalized to the total protein content of the sample, which was quantified using the bicinchoninic acid (BCA) protein assay kit (Pierce, Rockford, IL, USA) in accordance with the manufacturer’s instructions.

### Alizarin red staining and assay for the mineralized matrix

Cells were washed twice with cold PBS and fixed with 4% paraformaldehyde for 10 minutes. Then, they were stained with 30 mM Alizarin Red S (pH 4.2, Sigma) for 10 minutes at room temperature. then, images were obtained with a Canon camera. In order to quantify calcium deposition, Mineralization of calcium nodules were quantified by a method described previously^21^. Briefly, after staining, the cells were washed three times with PBS, 10% Hexadecylpyridinium Chloride Monohydrate (Sigma-Aldrich, Louis, MO, USA) was added and incubated for 20 mins at room temperature, then, Absorbance of the supernatant was measured at 540 nm in triplicate using ThermoMultiskan EX plate reader (Thermo Scientific, Waltham, MA, USA). Finally, the cells was washed with PBS and lysed with RIPA buffer and protein content was measured, the calcium levels were normalized to the total protein content.

### RNA isolation and real-time PCR

Total RNA was extracted from cells by TRizol reagent (Ambion, CA, USA) according to the manufacturer’s instructions. 2 μg total RNA was used for cDNA synthesis with Revert Aid First Strand cDNA Synthesis Kit (Thermo, Lithuania, EU). Real-time PCR in triplicate was performed with SYBR Green Master Mix (Applied Biosystems, Austin, TX, USA) on Applied Biosystems 7500 Real-Time PCR System as follows: 50 °C for two minutes, 95 °C for two minutes, and 40 cycles of 95 °C for 15 seconds and 60 °C for 60 seconds, The qPCR results were automatically analyzed using the Applied Biosystems 7500 system. The 2^−Δct^ method was used to calculate the relative gene expression. Primers of Real-time PCR was listed in Tables 2 and 3.

### Western blotting

Cell lysates was collected and total cell protein concentrations were determined by method described above. Equal amounts of protein were loaded on 10% sodium dodecyl sulfate-polyacrylamide gels. After electrophoresis, proteins were transferred to 0.2 μM Polyvinylidene fluoride membranes. Blots were blocked with 5% BSA (Amersco, Chengdu, China) at room temperature for one hour and incubated with the indicated primary antibodies overnight at 4 °C. After the blots were washed and incubated with HRP-conjugated secondary antibodies, blots were detected with enhanced chemiluminescence reagents (Millipore, Billerica, MA, USA) and quantified by densitometric analysis using the software Quantity One.

### Statistical analysis

All quantitative data are expressed as the means ± SD. Analysis was performed with SPSS 19.0. Comparisons between two groups were evaluated by the two-tailed Student’s t test. More than two groups differences were evaluated with a 2-way ANOVA followed by Tukey’s test. A level of P < 0.05 was considered statistically significant. Each experiment was repeated three times, and representative experiments are shown.

## Additional Information

**How to cite this article**: Xiaoyong, Q. *et al.* Irisin promotes osteoblast proliferation and differentiation via activating the MAP kinase signaling pathways. *Sci. Rep.* **6**, 18732; doi: 10.1038/srep18732 (2016).

## Supplementary Material

Supplementary Information

## Figures and Tables

**Table 1:** ALP Activity in signaling pathway experiment.

	Primary osteoblast (3 days)		MC3T3-L1 (3 days)	
PBS	191.57 ± 21.36		102.20 ± 11.55	
r-irisin	306.06 ± 21.45	P < 0.01*	193.10 ± 11.03	P < 0.01*
U0+ r-irisin	256.03 ± 10.02	P < 0.01#	137.33 ± 7.56	P < 0.01#
SB+ r-irisin	205.46 ± 18.58	P < 0.01#	117.50 ± 24.64	P < 0.01#

^*^Vs. PBS group.

^#^Vs. r-irisin group.

**Table 2:** The qPCR primers for Rat.

gene	Forward (5′-3′)	Reverse (5′-3′)
Runx_2_	TCATTCAGTGACACCACCAGG	TGTAGGGGCTAAAGGCAAAA
Osx	AGAAGCCATACACTGACCTTTC	GGTGGGTAGTCATTGGCATAG
ALP	GAGATGGTATGGGCGTCTC	GTTGGTGTTGTACGTCTTGGA
OC	GACAAGTCCCACACAGCAACT	GGACATGAAGGCTTTGTCAGA
OPG	CAAAGGCAGGGCATACTTC	TTCAATGATGTCCAAGAACACC
OPN	CCCATCTCAGAAGCAGAATCTT	GTCATGGCTTTCATTGGAGTTG
Col I 1α	GACATGTTCAGCTTTGTGGACCTC	GGGACCCTTAGGCCATTGTGTA
GAPDH	TATCGGACGCCTGGTTAC	CTGTGCCGTTGAACTTGC

**Table 3:** The qPCR primers for Mouse.

gene	Forward (5′-3′)	Reverse (5′-3′)
Runx_2_	CCGTGGCCTTCAAGGTTGT	TTCATAACAGCGGAGGCATTT
Osx	CCCTTCTCAAGCACCAATGG	AAGGGTGGGTAGTCATTTGCATA
ALP	TGACCTTCTCTCCTCCATCC	CTTCCTGGGAGTCTCATCCT
OC	CTTGAAGACCGCCTACAAAC	GCTGCTGTGACATCCATAC
OPG	GTGGAATAGATGTCACCCTGTGT	TTTGGTCCCAGGCAAACTGT
OPN	TGCACCCAGATCCTATAGCC	CTCCATCGTCATCATCATCG
Col I 1α	GCTCCTCTTAGGGGCCACT	CCACGTCTCACCATTGGGG
GAPDH	TGCACCACCAACTGCTTAG	GGATGCAGGGATGATGTTC
β-Actin	CAGCCTTCCTTCTTGGGTATG	GGCATAGAGGTCTTTACGGATG

## References

[b1] Varacallo M. A. & Fox E. J. Osteoporosis and its complications. Med Clin North Am 98, 817–831 (2014). 24994054 10.1016/j.mcna.2014.03.007

[b2] Golob A. L. & Laya M. B. Osteoporosis: screening, prevention, and management. Med Clin North Am 99, 587–606 (2015). 25841602 10.1016/j.mcna.2015.01.010

[b3] Oliveira A. & Vaz C. The role of sarcopenia in the risk of osteoporotic hip fracture. Clin Rheumatol 34, 1673–1680 (2015). 25912213 10.1007/s10067-015-2943-9

[b4] Papaioannou A. *et al.* clinical practice guidelines for the diagnosis and management of osteoporosis in Canada: summary. CMAJ 182, 1864–1873 (2010). 20940232 10.1503/cmaj.100771 PMC2988535

[b5] Cosman F. *et al.* Clinician’s Guide to Prevention and Treatment of Osteoporosis. Osteoporos Int 25, 2359–2381 (2014). 25182228 10.1007/s00198-014-2794-2 PMC4176573

[b6] DiGirolamo D. J., Kiel D. P. & Esser K. A. Bone and skeletal muscle: neighbors with close ties. J Bone Miner Res 28, 1509–1518 (2013). 23630111 10.1002/jbmr.1969 PMC4892934

[b7] Lang D. H. *et al.* Bone, muscle, and physical activity: structural equation modeling of relationships and genetic influence with age. J Bone Miner Res 24, 1608–1617 (2009). 19419307 10.1359/JBMR.090418 PMC2730930

[b8] Nordstrom A. *et al.* Bone loss and fracture risk after reduced physical activity. J Bone Miner Res 20, 202–207 (2005). 15647813 10.1359/JBMR.041012

[b9] Pedersen B. K. & Febbraio M. A. Muscles, exercise and obesity: skeletal muscle as a secretory organ. Nat Rev Endocrinol 8, 457–465 (2012). 22473333 10.1038/nrendo.2012.49

[b10] Elkasrawy M. N. & Hamrick M. W. Myostatin (GDF-8) as a key factor linking muscle mass and bone structure. J Musculoskelet Neuronal Interact 10, 56–63 (2010). 20190380 PMC3753581

[b11] Bostrom P. *et al.* A PGC1-alpha-dependent myokine that drives brown-fat-like development of white fat and thermogenesis. Nature 481, 463–468 (2012). 22237023 10.1038/nature10777 PMC3522098

[b12] Zhang W. *et al.* Irisin: A myokine with locomotor activity. Neurosci Lett 595, 7–11 (2015). 25841790 10.1016/j.neulet.2015.03.069 PMC4836606

[b13] Huh J. Y., Dincer F., Mesfum E. & Mantzoros C. S. Irisin stimulates muscle growth-related genes and regulates adipocyte differentiation and metabolism in humans. Int J Obes (Lond) 38, 1538–1544 (2014). 24614098 10.1038/ijo.2014.42

[b14] Hofmann T., Elbelt U. & Stengel A. Irisin as a muscle-derived hormone stimulating thermogenesis–a critical update. Peptides 54, 89–100 (2014). 24472856 10.1016/j.peptides.2014.01.016

[b15] Park K. H. *et al.* Circulating irisin in relation to insulin resistance and the metabolic syndrome. J Clin Endocrinol Metab 98, 4899–4907 (2013). 24057291 10.1210/jc.2013-2373 PMC3849667

[b16] Palermo A. *et al.* Irisin is associated with osteoporotic fractures independently of bone mineral density, body composition or daily physical activity. Clin Endocrinol (Oxf) 82, 615–619 (2015). 25400208 10.1111/cen.12672

[b17] Anastasilakis A. D. *et al.* Circulating irisin is associated with osteoporotic fractures in postmenopausal women with low bone mass but is not affected by either teriparatide or denosumab treatment for 3 months. Osteoporos Int 25, 1633–1642 (2014). 24599275 10.1007/s00198-014-2673-x

[b18] Colaianni G. *et al.* Irisin enhances osteoblast differentiation *in vitro*. Int J Endocrinol 2014, 902186 (2014). 24723951 10.1155/2014/902186 PMC3960733

[b19] Albrecht E. *et al.* Irisin - a myth rather than an exercise-inducible myokine. Sci Rep 5, 8889 (2015). 25749243 10.1038/srep08889 PMC4352853

[b20] Zhang Y. *et al.* Irisin stimulates browning of white adipocytes through mitogen-activated protein kinase p38 MAP kinase and ERK MAP kinase signaling. Diabetes 63, 514–525 (2014). 24150604 10.2337/db13-1106 PMC13117908

[b21] Luo X. H. *et al.* Adiponectin stimulates human osteoblasts proliferation and differentiation via the MAPK signaling pathway. Exp Cell Res 309, 99–109 (2005). 15963981 10.1016/j.yexcr.2005.05.021

[b22] Lian J. B.& Stein G. S. The developmental stages of osteoblast growth and differentiation exhibit selective responses of genes to growth factors (TGF beta 1) and hormones (vitamin D and glucocorticoids). J Oral Implantol 19, 95–105; discussion 136–107 (1993). 8246305

[b23] Stein G. S. & Lian J. B. Molecular mechanisms mediating proliferation/differentiation interrelationships during progressive development of the osteoblast phenotype. Endocr Rev. 14, 424–442 (1993). 8223340 10.1210/edrv-14-4-424

[b24] Lian J. B. *et al.* Regulatory controls for osteoblast growth and differentiation: role of Runx/Cbfa/AML factors. Crit Rev Eukaryot Gene Expr. 14, 1–41 (2004). 15104525

[b25] Lian J. B. & Stein G. S. Development of the osteoblast phenotype: molecular mechanisms mediating osteoblast growth and differentiation. Iowa Orthop J. 15, 118–140 (1995). 7634023 PMC2329080

[b26] Lian J. B. & Stein G. S. Concepts of osteoblast growth and differentiation: basis for modulation of bone cell development and tissue formation. Crit Rev Oral Biol Med. 3, 269–305 (1992). 1571474 10.1177/10454411920030030501

[b27] Sundell J. Resistance Training Is an Effective Tool against Metabolic and Frailty Syndromes. Adv Prev Med 2011, 984683 (2011). 21991450 10.4061/2011/984683 PMC3168930

[b28] Moon H. S., Dincer F. & Mantzoros C. S. Pharmacological concentrations of irisin increase cell proliferation without influencing markers of neurite outgrowth and synaptogenesis in mouse H19-7 hippocampal cell lines. Metabolism 62, 1131–1136 (2013). 23664146 10.1016/j.metabol.2013.04.007 PMC4370428

[b29] Song H. *et al.* Irisin promotes human umbilical vein endothelial cell proliferation through the ERK signaling pathway and partly suppresses high glucose-induced apoptosis. PLoS One 9, e110273 (2014). 25338001 10.1371/journal.pone.0110273 PMC4206299

[b30] Colaianni G. *et al.* The myokine irisin increases cortical bone mass. Proc Natl Acad Sci USA 112, 12157–12162 (2015). 26374841 10.1073/pnas.1516622112 PMC4593131

[b31] Vaughan R. A. *et al.* Characterization of the metabolic effects of irisin on skeletal muscle *in vitro*. Diabetes Obes Metab 16, 711–718 (2014). 24476050 10.1111/dom.12268

[b32] Zeng W., Yan Y., Z.hang F., Zhang C. & Liang W. Chrysin promotes osteogenic differentiation via ERK/MAPK activation. Protein & cell 4, 539–547 (2013). 23744338 10.1007/s13238-013-3003-3 PMC4875509

[b33] Wang X. *et al.* Spata4 promotes osteoblast differentiation through Erk-activated Runx2 pathway. J Bone Miner Res 26, 1964–1973 (2011). 21445983 10.1002/jbmr.394

[b34] Hou X. *et al.* A specific oligodeoxynucleotide promotes the differentiation of osteoblasts via ERK and p38 MAPK pathways. Int J Mol Sci. 13, 7902–7914 (2012). 22942680 10.3390/ijms13077902 PMC3430211

[b35] Srivastava S., Kumar N. & Roy P. Role of ERK/NFkappaB in vanadium (IV) oxide mediated osteoblast differentiation in C3H10t1/2 cells. Biochimie 101, 132–144 (2014). 24440756 10.1016/j.biochi.2014.01.005

[b36] Liu J. Irisin as an exercise-stimulated hormone binding crosstalk between organs. Eur Rev Med Pharmacol Sci 19, 316–321 (2015). 25683949

[b37] Schumacher M. A., Chinnam N., Ohashi T., Shah R. S. & Erickson H. P. The structure of irisin reveals a novel intersubunit beta-sheet fibronectin type III (FNIII) dimer: implications for receptor activation. J Biol Chem 288, 33738–33744 (2013). 24114836 10.1074/jbc.M113.516641 PMC3837118

[b38] Daskalopoulou S. S. *et al.* Plasma irisin levels progressively increase in response to increasing exercise workloads in young, healthy, active subjects. Eur J Endocrinol 171, 343–352 (2014). 24920292 10.1530/EJE-14-0204

[b39] Kim H. J., So B., Choi M., Kang D. & Song W. Resistance exercise training increases the expression of irisin concomitant with improvement of muscle function in aging mice and humans. Exp Gerontol 70, 11–17 (2015). 26183690 10.1016/j.exger.2015.07.006

[b40] Hew-Butler T. *et al.* Plasma irisin in runners and nonrunners: no favorable metabolic associations in humans. Physiol Rep 3 (2015). 10.14814/phy2.12262 PMC4387758 25602017

[b41] Huh J. Y. *et al.* FNDC5 and irisin in humans: I. Predictors of circulating concentrations in serum and plasma and II. mRNA expression and circulating concentrations in response to weight loss and exercise. Metabolism 61, 1725–1738 (2012). 23018146 10.1016/j.metabol.2012.09.002 PMC3614417

[b42] Huh J. Y., Mougios V., Skraparlis A., Kabasakalis A. & Mantzoros C. S. Irisin in response to acute and chronic whole-body vibration exercise in humans. Metabolism 63, 918–921 (2014). 24814685 10.1016/j.metabol.2014.04.001

[b43] Moraes C. *et al.* Resistance exercise training does not affect plasma irisin levels of hemodialysis patients. Horm Metab Res 45, 900–904 (2013). 24013946 10.1055/s-0033-1354402

[b44] Kerr D., Ackland T., Maslen B., Morton A. & Prince R. Resistance training over 2 years increases bone mass in calcium-replete postmenopausal women. J Bone Miner Res 16, 175–181 (2001). 11149482 10.1359/jbmr.2001.16.1.175

[b45] Orriss I. R., Hajjawi M. O., Huesa C., MacRae V. E. & Arnett T. R. Optimisation of the differing conditions required for bone formation *in vitro* by primary osteoblasts from mice and rats. Int J Mol Med 34, 1201–1208 (2014). 25200658 10.3892/ijmm.2014.1926 PMC4199408

[b46] Vater C., Kasten P. & Stiehler M. Culture media for the differentiation of mesenchymal stromal cells. Acta Biomater 7, 463–477 (2011). 20688199 10.1016/j.actbio.2010.07.037

