# Corrigendum: Irisin promotes osteoblast proliferation and differentiation via activating the MAP kinase signaling pathways

**DOI:** 10.1038/srep21053

**Published:** 2016-02-15

**Authors:** Xiaoyong Qiao, Ying Nie, Yaxian Ma, Yan Chen, Ran Cheng, Weiyao Yin, Ying Hu, Wenming Xu, Liangzhi Xu

Scientific Reports 6: Article number: 18732; 10.1038/srep18732 published online: 01072016; updated: 02152016

The original version of this Article contained typographical errors in the spelling of the authors Xiaoyong Qiao, Yaxian Ma, Weiyao Yin, Wenming Xu and Liangzhi Xu which were incorrectly given as Xiao Yong Qiao, Ya Xian Ma, Wei Yao Yinrg, Wen Ming Xu and Liang Zhi Xu respectively.

In addition, there was a typographical error in the Abstract.

“Inhibition of p38 MAPK by SB023580 or pERK by U0126 abolished the proliferation and up-regulatory effects of irisin on Runx_2_ expression and ALP activity.”

now reads

“Inhibition of p38 MAPK by SB203580 or pERK by U0126 abolished the proliferation and up-regulatory effects of irisin on Runx_2_ expression and ALP activity.”

These errors have now been corrected in the PDF and HTML versions of the Article.

