# Supplementary material for: Irisin promotes osteoblast proliferation and differentiation via activating the MAP kinase signaling pathways
Source: Sci Rep. 2016 Jan 7;6:18732. doi: 10.1038/srep18732 (PMC4704023; doi:10.1038/srep18732)
Supplement: Supplementary Information [file srep18732-s1.pdf]

Irisin promotes osteoblast proliferation and differentiation via activating the MAP kinase signaling pathways

Xiao Yong Qiao[1,2,&], Ying Nie[1,2,&], Ya Xian Ma[1,2], Yan Chen[1,2], Ran Cheng[1,2], Wei Yao Ying[1,2], Ying Hu[1,2], Wen Ming Xu[2], Liang Zhi Xu[1,2,]

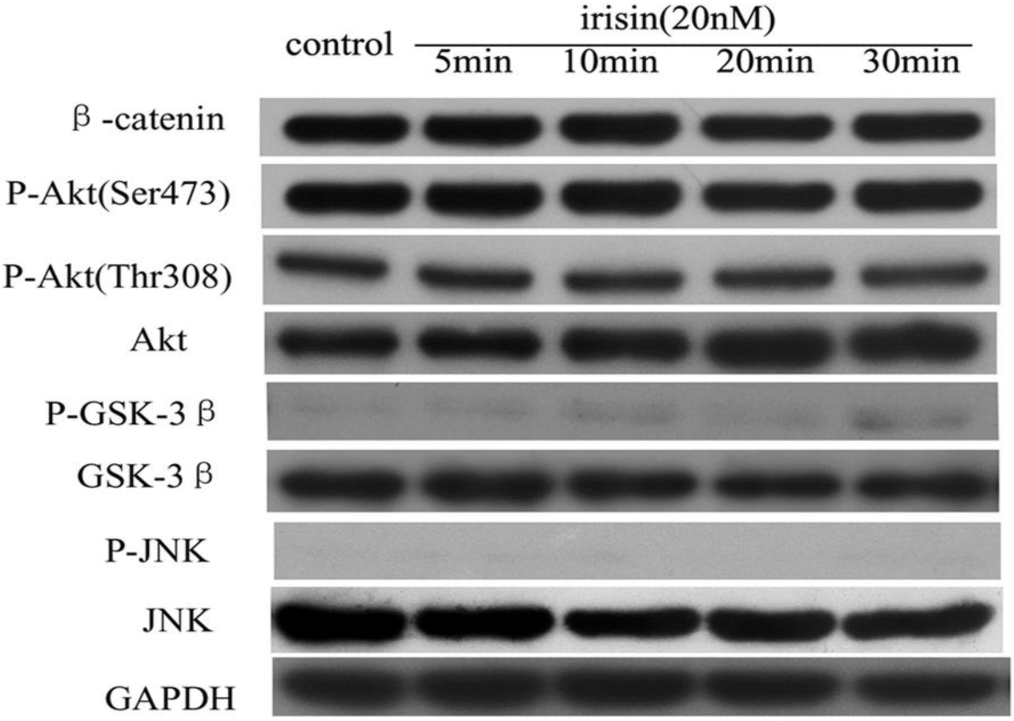

Supplementary Figure 1. The canonical WNT, AKT, and JNK signaling pathways not involved in irisin-induced osteogenic effect. Representative western blotting images of p-Akt (Ser473), p-Akt (Thr308), p-JNK, and p-GSK-3β and the total protein of Akt, JNK, GSK-3β, β-catenin, and GAPDH. The phosphorylated proteins levels and the amount of total proteins levels of these pathways were unchanged after treatment with r-irisin (20nM) at the indicated time.

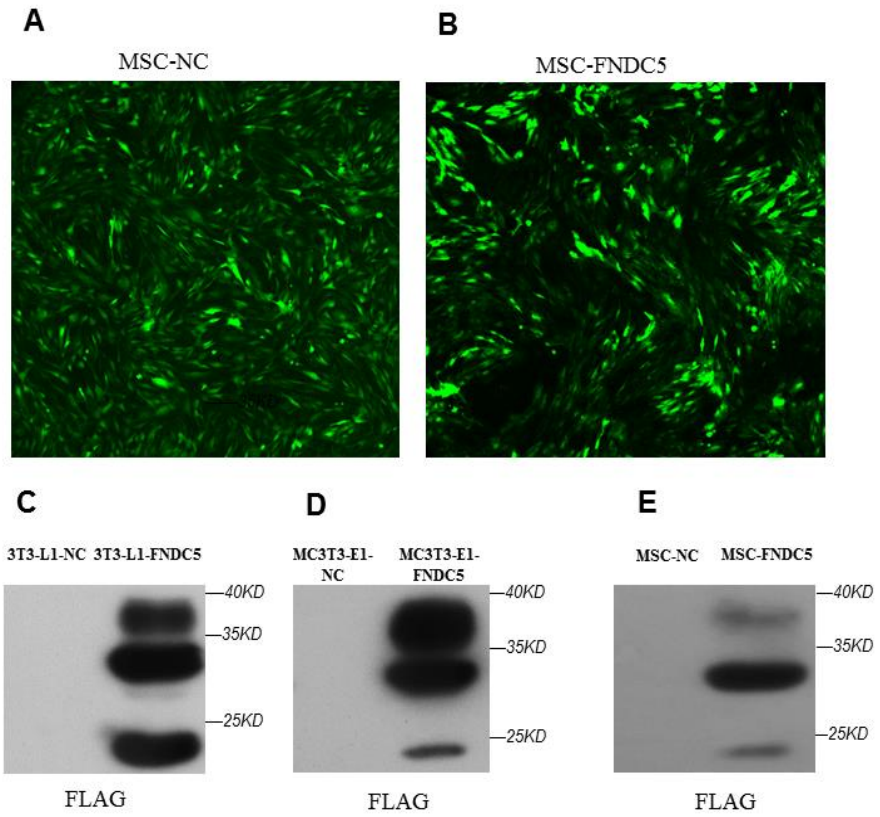

Supplementary Figure 2. FNDC5 transfection and FLAG identification. A. representative images of MSC-FNDC5 cells (mouse bone marrow mesenchymal stem cells transfected with FNDC5 lentivirus and stable express FNDC5-flag and GFP)and MSC-NC cells (mouse bone marrow mesenchymal stem cells transfected with negative control lentivirus，and stable express GFP) B The FLAG protein expression in 3T3-L1-NC, 3T3-L1-FNDC5, MC3T3-E1-NC, MC3T3-E1-FNDC5, MSC-NC and MSC-FNDC5 cell lysates were analyzed by Western blotting.

Supplementary Table 1. OD450nm of osteoblast proliferation curve

	MC3T3-E1			Primary osteoblast		
	**CM-control**	**CM-irisin**	**P**	**CM-control**	**CM-irisin**	**P**
**0**	0.139±0.007	0.137±0.004	0.415	0.141±0.006	0.137±0.005	0.273
**24h**	0.417±0.021	0.479±0.018	<0.01	0.344±0.011	0.382±0.019	<0.01
**48h**	1.120±0.067	1.314±0.071	<0.01	0.601±0.026	0.714±0.037	<0.01
**72h**	2.174±0.116	2.688±0.227	<0.01	1.176±0.081	1.510±0.073	<0.01

Supplementary Table 2. OD450nm of primary osteoblast proliferation curve

	PBS	r-irisin	U0+ r-irisin	SB+ r-irisin
**0**	0.136±0.008	0.138±0.013	0.140±0.011	0.137±0.007
**24h**	0.245±0.008	0.269±0.014*	0.238±0.009#	0.240±0.008#
**48h**	0.527±0.037	0.637±0.026*	0.482±0.046#	0.499±0.065#
**72h**	1.142±0.085	1.411±0.095*	1.017±0.071#	0.971±0.084#

*: $P<0.05$ Vs. PBS group; #: $P<0.05$ Vs. r-irisin group.

Supplementary Table 3. OD450nm of MC3T3-E1 proliferation curve

	PBS	r-irisin	U0+ r-irisin	SB+ r-irisin
**0**	0.145±0.006	0.151±008	0.148±0.009	0.147±0.011
**24h**	0.375±0.013	0.391±0.009*	0.375±0.009#	0.379±0.012#
**48h**	1.007±0.082	1.212±0.037*	0.990±0.074#	1.001±0.103#
**72h**	1.998±0.069	2.420±0.126*	1.846±0.152#	1.825±0.104#

*: $P<0.05$ Vs. PBS group; #: $P<0.05$ Vs. r-irisin group.

Supplementary Table 4. OD450nm of osteoblast proliferation curve

	MC3T3-E1-NC	MC3T3-E1-FNDC5	P
**0**	0.320±0.041	0.324±0.028	0.415
**24h**	0.563±0.021	0.656±0.063	0.09
**48h**	0.724±0.082	0.918±0.106	<0.01
**72h**	1.246±0.083	1.516±0.121	<0.01

Supplementary Table 5. ALP activity after osteogenic culture for 14days

	PBS	r-irisin	CM-control	CM-irisin
**Primary osteoblast**	268.54±24.29	351.47±41.63*	368.30±29.36	535.69±66.26#
**MC3T3-E1**	182.65±13.78	283.06±20.65*	223.20±35.23	399.88±49.25#

*: P<0.05 Vs. PBS group; #: P<0.05 Vs. CM-control group.
